# Determinants of Double Burden of Malnutrition Among School Children and Adolescents in Urban Dhaka: A Multi-Level Analyses

**DOI:** 10.3389/fpubh.2022.926571

**Published:** 2022-07-15

**Authors:** Md. Tariqujjaman, Sifat Parveen Sheikh, George Smith, A. M. Rumayan Hasan, Fatema Khatun, Ashraful Kabir, Md. Harunor Rashid, Sabrina Rasheed

**Affiliations:** ^1^Nutrition and Clinical Services Division, International Center for Diarrhoeal Disease Research, Bangladesh (icddr, b), Dhaka, Bangladesh; ^2^Health Systems and Population Studies Division, International Center for Diarrhoeal Disease Research, Bangladesh (icddr, b), Dhaka, Bangladesh; ^3^Fragments Magazine, Dhaka, Bangladesh; ^4^School of Public Health and Preventive Medicine, Monash University, Melbourne, VIC, Australia

**Keywords:** double burden of malnutrition, school children, adolescent, urban Bangladesh, LMIC, underweight, overweight, obesity

## Abstract

**Background:**

Bangladesh faces a double burden of malnutrition, with a rising prevalence of overweight and obesity among children and adolescents parallel to existing undernutrition.

**Objective:**

The current study was designed to assess the determinants of double burden of malnutrition among urban school children and adolescents from Dhaka, Bangladesh.

**Methods:**

A cross-sectional survey was conducted among 2,690 students from 14 schools in Dhaka city from January to June 2018. Anthropometric measurements were taken during school hours, and self-administered questionnaires were sent to the parents. We performed multi-level multiple logistic regression analyses to assess the determinants of underweight, overweight, and obesity.

**Findings:**

The prevalence of overweight (33%) and obesity (23%) was highest among children and adolescents from high tuition schools but the prevalence of underweight (4%) was lowest compared to those from low (underweight 19%, overweight 17%, and obesity 6%) and medium (underweight 18%, overweight 15%, and obesity 6%) tuition schools. Children and adolescents from high-tuition schools had higher odds of being overweight/ obese (AOR: 2.92; 95% CI: 1.90, 4.49). Parental NCDs and overweight were negatively associated with underweight but positively associated with overweight and obesity among children and adolescents. Lack of physical activity inside schools was positively associated (AOR: 1.26; 95% CI: 1.02, 1.55) with overweight and obesity among school children and adolescents.

**Conclusion:**

Our results point to opportunities in and outside schools to address the rising prevalence of underweight, overweight, and obesity among urban school children and adolescents.

## Introduction

In 2016, there were over 340 million children and adolescents with overweight or obesity around the world (1). Globally, from 1975 to 2016, the prevalence of overweight and obesity among 5–19 years old increased from 4 to 18% (1). This increase has been 30% higher in low-and middle-income countries (LMICs) compared to high-income countries (HICs) and a majority of children with overweight or obesity now live in LMICs (2). Historically in LMICs, underweight among children has been more prevalent. However, overweight and obesity is on the rise with rapid urbanization linked to increased consumption of high-calorie processed food and sedentary lifestyles (3). This double burden of malnutrition presents a wide range of problems (4). Children and adolescents who are underweight are more likely to have poor cognition, nutritional deficiencies and are more susceptible to infections (4). Studies have shown that poor health and nutritional status among adolescent girls leads to the intergenerational cycle of malnutrition, low productivity, and economic losses (5). On the other hand, children who are overweight or obese before puberty are more likely to retain their high body weight into early adulthood (6) and develop non-communicable diseases (NCDs) such as hypertension, ischemic heart disease, dyslipidemia, type 2 diabetes, gallbladder diseases, osteoarthritis and different types of cancer (7). In Bangladesh, NCDs account for about 41% of the total disease burden and about 51% of mortality, annually (8). Increased prevalence of overweight and obesity in addition to the existing burden of underweight among children and adolescents can potentially lower life expectancy and drive up healthcare costs (9).

Despite the growing double burden of malnutrition in Bangladesh, only a handful of studies have assessed underweight and overweight/ obesity among school children and adolescents. In a study conducted among 6–12 year old Bangladeshi children researchers reported prevence of overweight and obesity to be 10% and 5% respectively whereas the prevelance of underweight was between 16.3% and 12.7% among boys and girls respectively (10). The children living in poor households were significantly more likely to be underweight compared to others whereas children from better off households were more likely to be overweight or obese compared to others (10). In 2014, a national survey researchers reported 9.5% overweight and 3.5% obesity among 6–15-year-old children and adolescents (11). In terms of risk factors, male sex, higher socioeconomic status, residing in urban non-slum areas, spending more than 4 h per day in sedentary activities and having a parent with overweight were found to be associated with a greater risk of obesity among 10–17-year-old school children and adolescents (12). For underweight, having less educated parents, inadequate food intake, poor hygiene practices were significant risk factors among school children in urban informal settlements (13). Despite the growing evidence on the double burden of malnutrition among school children and adolescents, there is a paucity of literature that explore the related risk factors. Moreover, a study (14) that assessed the double burden of malnutrition among school children in Bangladesh have not adopted a conceptual framework to assess a wide range of modifiable risk factors. Further, most studies (10, 12) on malnutrition among school children do not include a wide variety of urban schools, so the results may not be applicable to children attending different types of schools in urban areas. Our study aims to address the gap in the existing literature by including children and adolescents from different tuition categories (high, medium, low) of schools and by using an existing conceptual framework to examine a range of modifiable risk factors that are associated with both underweight and overweight/obesity among urban school children (15). Understanding the risk factors will help inform and design interventions that focus on high-risk groups.

## Methods

### Study Design and Participants

We conducted a cross-sectional survey among students aged 6–15 years from 14 schools in Dhaka city. The survey was conducted from January to June 2018. The schools were randomly selected from both administrative areas of Dhaka: Dhaka North and South. For schools selected, a list of students aged 6–15 years was obtained. The number of students was stratified by gender and randomly selected to ensure equal distribution. The final study sample consisted of 2,690 students. The height and weight of the students were measured on school premises by trained field workers. The standing height for each student was measured and recorded to the nearest 0.1 cm using a stadiometer. Body weights were measured on SECA digital scales to the nearest 0.1 kg. All measurements were made with the children wearing light clothes and no footwear, and the scales were adjusted to “0” before each measurement to ensure accuracy. After recording the anthropometric measurements, a pre-tested, self-administered questionnaire was given to the students for their parents to complete. The research team collected the questionnaires from the teachers on a designated day a week after they were given out.

### Sample Size and Sampling Technique

After the initial mapping of all schools in the chosen areas, the schools were stratified into low, medium and high tuition schools.

In this study, we calculated the sample size considering a 5.6% prevalence of obesity (11) with 2.8% precision, 95% confidence level, 10% non-response rate, and design effect of 2. Our minimum estimated sample size for each type of school (low, medium, or high tuition) of an area (Dhaka north or Dhaka south city corporation) was 572.

A multistage stratified sampling technique was applied to select the students in each class. First, we purposively selected one police station/ thana from each of the two administrative areas of Dhaka: Dhaka North and Dhaka South. We then listed all schools in these two areas and included the schools which had enrolled students from class 1 to class 10. We categorized the school types as low, medium, and high-tuition schools by totaling the school tuition and admission fees and categorized as USD 4–24 low-tuition USD 25–70 as medium-tuition, and USD 71–276 as high-tuition schools. From each stratum, schools were randomly selected. During our initial random selection, 6 schools (2 low, 2 medium, and 2 high tuition schools) were chosen for each thana. For each girls' school selected an additional boys' school was chosen from the list to ensure an equal male: female ratio in the study sample. The final selection consisted of 14 schools (Table 1). To reach the estimated sample size, 30 students (15 boys and 15 girls) from each class were required to participate in our study. The total sample size for each area for all three school types was 30^*^10^*^6^*^3 = 1,800. The total required sample size for the two sites was 3,600. The total number of students present in our selected schools was 11,861. Among them, we randomly selected 3,600 students (30 for each class). Of the selected participants, 910 opted out from the survey. Finally, we included the information of 2,690 participants (response rate 75%) in our final analysis.

**Table 1:** Characteristics of schools.

**Characteristics**	**Tuition Fee**		
	**High**	**Medium**	**Low**
**Administrative areas**			
Dhaka north	2	2	3
Dhaka south	2	2	3
**Medium of instruction**			
English only	3	0	0
Bengali/English	1	0	0
Bengali only	0	4	6
**Gender-based classification**			
Co-education	4	4	4
Single sex	0	0	2

### Outcome Variable

The outcome variable for this study was the Body Mass Index (BMI). Age and sex-specific overweight, obesity, and underweight based on BMI-for-age z-scores were estimated using the World Health Organization (WHO) BMI cut-off points. To determine the underweight, normal weight, overweight, and obesity, BMI-for-age z-score −3 to −1, 0, +1, and +2 respectively were used as cut-off points (16). The *zanthro* package of Stata software was used to calculate the BMI-for-age z-score. In the regression analyses, we merged overweight and obesity due to the low prevalence of obesity.

### Independent Variables

**Figure 1:**
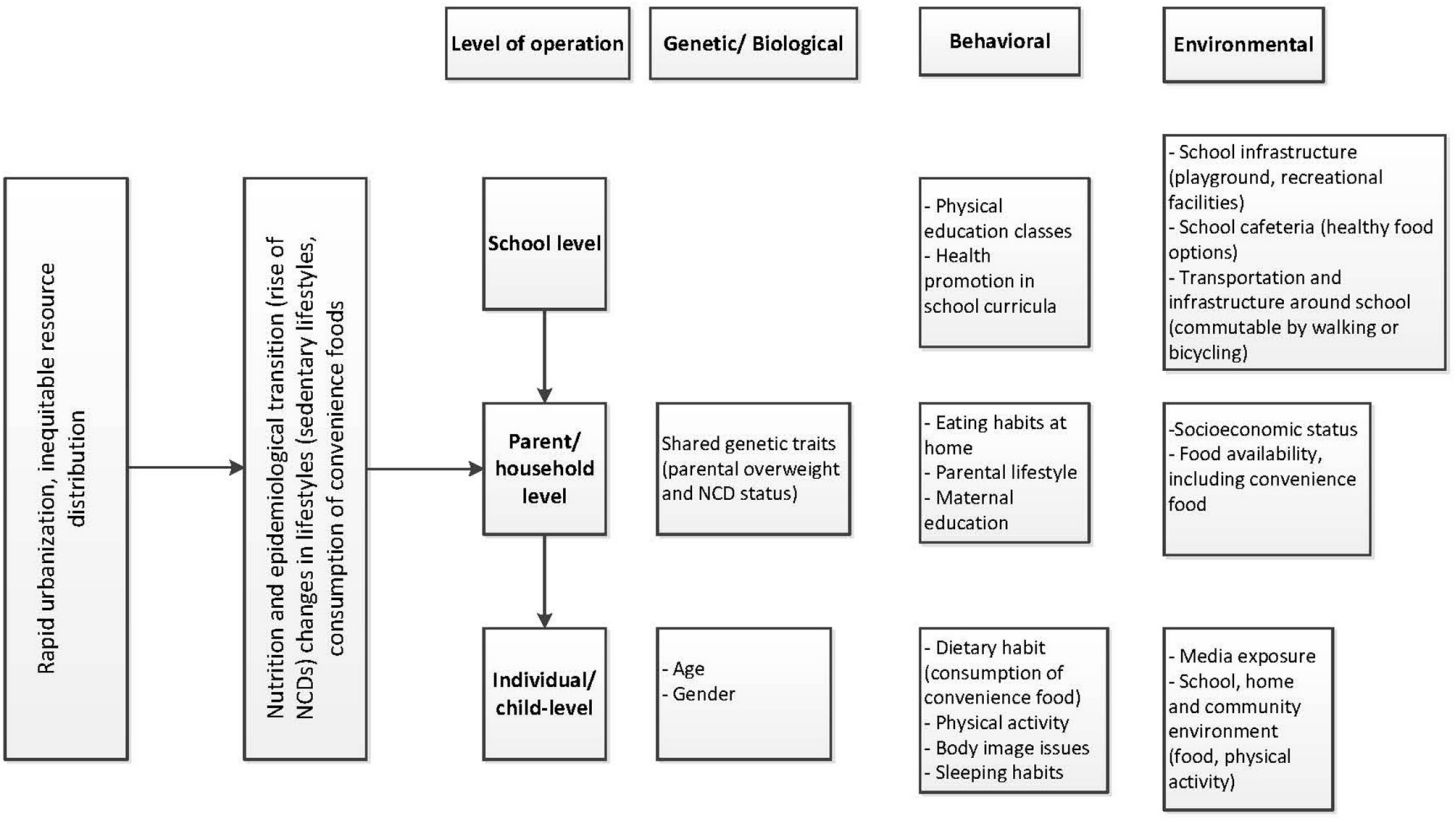
Framework conceptualizing the modifiable risk factors of childhood and adolescent overweight and obesity. Note: The framework is adapted from Kimani-Murage, (15) Conceptual framework on nutrition transition and hierarchical organization of factors influencing a child's nutritional status (15).

A validated and pre-tested tool was used to collect information on multiple independent variables based on themes from an existing conceptual framework (Figure 1) which stipulates that socioeconomic status, lifestyle related to physical activity and diet, sleep patterns, and parental determinants are some of the modifiable risk factors that affect overweight and obesity and underweight (15). For our study, child-level determinants included age (<10, 10-13, and ≥14 years), sex (male, female), feeding index (unhealthy and healthy), physical activity, skip breakfast (yes, no), and duration of sleep per 24h (≥8 hours, <8 h). The feeding index variable was constructed using healthy and unhealthy feeding practices. The unhealthy practices included the frequency of fast food (fried chicken, sauces, Burger, Shwarma) and beverages (cold drinks/ juice) consumption in a week (never, 1–4 times and 5 or more times). Based on frequency of consumption of these unhealthy foods and drinks, individual respondents got a score of 0 to −2 (0 = never consumed, −1 = 1–4 times consumed, and −2 = 5 or more times). In the case of healthy practices, we included the frequencies of vegetables and fruit consumption in the last week. Based on the frequency of consumption of fruits and vegetables, individual respondents got a score of 0 to 2 (0 = never, 1 = 1–4 times, and 2 = 5 or more times). Then we summed up both the unhealthy and healthy feeding scores. Finally, we categorized it as unhealthy (−4 to 0) and healthy (0 to 4) feeding index. To assess the physical activity levels of the child, the “walking and bicycling” variable was constructed based on a question about whether the child walked or bicycled. If the response was “yes” for both questions, we classified it as “yes,” and all other responses were coded as “no”. The average time of daily walking or bicycling was 80 min. The school-level characteristics included school type based on the tuition fees (low, medium, and high), and participation in school physical activities (yes, no). The parental determinants included parental education (0–5, 6–10, and >10 years of schooling), mother's employment (yes, no), parental non-communicable disease status (hypertension, diabetes, asthma, and other self-reported NCDs) categorized as none, one parent with NCDs, both parents with NCDs and self-reported parental weight categorized as normal weight, one overweight parent, both overweight parents.

### Statistical Analyses

We performed descriptive analysis and presented the estimates in percentages with frequencies. We applied multi-level logistic regression analysis to assess the determinants of underweight and overweight/obese due to the hierarchal nature of the data. In our study, the study children were nested in the classes and further the classes were nested within schools. The multi-level mixed effect models have the potential to capture multiple hierarchal data structures and to reduce the problem of overestimation (17). To adjust the intraclass correlation, we applied a three-level logistic regression model with the random intercept at school and class levels. We ran three separate models for underweight and overweight/obese, respectively. The first model included children-level characteristics. The second model included both children and household-level characteristics. The final model included children, household, and school-level characteristics. We presented the estimates in odds ratios with respective 95% confidence intervals. We ran collinearity diagnostics to test for collinearity among independent variables. The mean variance inflation factor for all the variables was <1.5, indicating no multicollinearity within the independent variables in the models. The statistical software package Stata Version 15.0 was used for data analyses.

### Ethics Considerations

Ethical clearance for this study was obtained from the Ethical Review Committee of the International Center for Diarrhoeal Disease Research, Bangladesh (icddr, b) (Protocol Number: PR-18021). All parents provided informed written consent for participating in this study and for recording the BMI of their children. In addition, we obtained written consent from the school authorities, verbal assent from the students aged 6–10 years, and written assent from the students aged 11–15 years. Each of the students was assigned an ID number to maintain anonymity.

## Results

### Sample Characteristics

The anthropometric measurements from 2,690 school-going children and adolescents were available for analysis. Among the participants, 41% were from low-tuition schools, 42% from medium-tuition schools, and 16.8% from high tuition schools. Among parental characteristics, 38.8% of the fathers of the study participants had more than 10 years of schooling, while 27.2% of mothers had more than 10 years of schooling. About 20% of the mothers of the study participants were employed. For 16.6% of the study participants, both parents suffered from NCDs, and in 6.8%, both parents were overweight. In terms of school physical activities, about 61% of students participated in some form of school sports and about 12% walked and bicycled to school (Table 2).

**Table 2:** Characteristics of the study participants (*n* = 2,690).

**Characteristics**	***n***	***Proportion (%)***
Child characteristics		
**Age of child (y)**		
<10	1,137	42.3
10–13	1,103	41.0
≥14	450	16.7
**Sex**		
Female	1,239	46.0
Male	1,451	54.0
**Skip breakfast**		
No	1,110	41.3
Yes	1,580	58.7
Feeding index		
Unhealthy	1,154	43.6
Healthy	1,494	56.4
**Walking and bicycling**		
Yes	330	12.3
No	2,360	87.7
**Sleep duration (/24 h) (*****n*** **= 2574)**		
≥8	1,917	74.5
<8	657	25.5
Parental characteristics		
**Father's education (y)**		
0–5	778	28.9
6–10	869	32.3
>10	1,043	38.8
**Mother's education (y)**		
0–5	839	31.2
6–10	1,119	41.6
>10	732	27.2
**Mother's employment**		
No	2,142	79.6
Yes	548	20.4
**Parental NCDs**		
No parent with NCD	1,309	48.7
One parent with NCD	935	34.8
Both parents with NCDs	446	16.6
**Parental weight**		
Normal weight	1,784	66.3
One parent overweight	722	26.8
Both parents overweight	184	6.8
**School characteristics**		
**School type**		
Low tuition	1,094	40.7
Medium tuition	1,143	42.5
High tuition	453	16.8
**Participation in school P.A**.		
Yes	1,648	61.3
No	1,042	38.7

*PA, Physical Activity*.

### Prevalence of Underweight and Overweight/Obesity Among School Children

**Figure 2:**
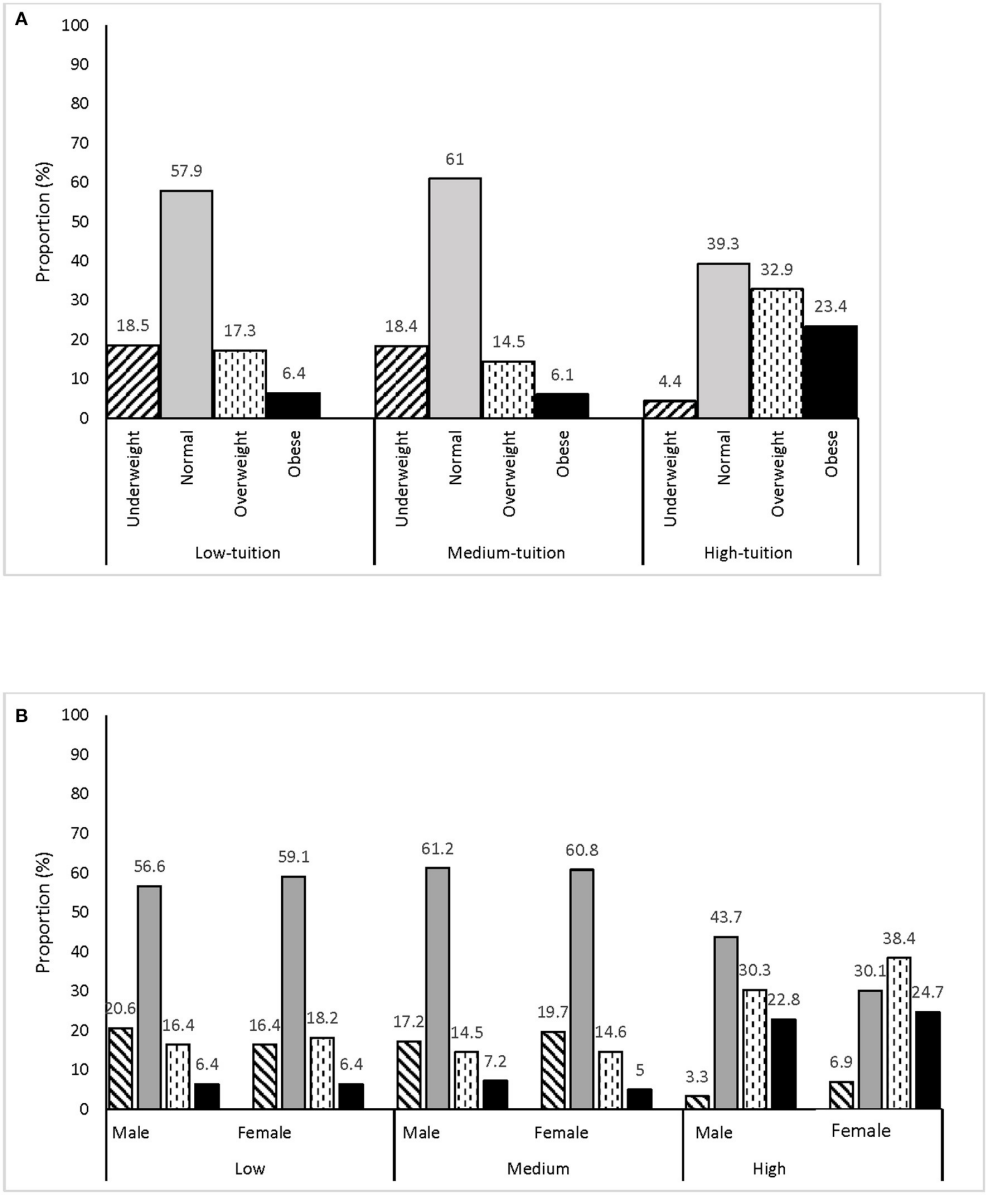
Weight status among school children by school category **(A)** and gender **(B)**.

Overall the prevalence of underweight, normal, overweight, and obesity were 16, 56, 19, and 9%, respectively (not shown in the table or figure). The prevalence of overweight (33%) and obesity (23%) was highest among students in high-tuition schools (Figure 2A) with this overweight trend largely driven by the female students (Figure 2B). Noteworthy is the prevalence of underweight in all schools, which aptly represents the double burden of malnutrition (Figure 2A).

### Association of Underweight and Overweight/Obesity With Sample Characteristics

In the bivariate analysis, we found that high-tuition school, older age of the child, mothers with >10 years of schooling, both parents having NCDs, one and both parents being overweight, skip breakfast, and sleep duration of <8 h were significantly associated with lower odds of the child being underweight. On the other hand, high-tuition schools, one and both parents having NCDs, one and both parents being overweight, and mothers with >10 years of schooling were significantly associated with higher odds of overweight and obesity (Table 3).

**Table 3:** Association between underweight and overweight/obesity with background characteristics.

**Characteristics**	**Normal weight**	**Underweight**	***p*-value**	**Normal vs**.	**Normal**	**Overweight/Obese**	***p*-value**	**Normal vs**.
	***n* = 1,508**	***n* = 432**		**underweight**	**weight**	***n* = 253**		**overweight/obese**
		***n* (%)**		**OR (95% CI)**		***n* (%)**		**OR (95% CI)**
**Child characteristics**								
**Age of child (y)**								
<10	600 (71.8)	236 (28.2)	<0.001	Ref.	600 (66.6)	301 (33.4)	0.086	Ref.
10–13	629 (82.1)	137 (17.9)		0.56*** (0.43, 0.74)	629 (65.1)	337 (34.9)		1.07 (0.85, 1.35)
≥14	279 (82.5)	59 (17.5)		0.54** (0.37, 0.77)	279 (71.4)	112 (28.6)		0.98 (0.72, 1.33)
**Sex**								
Female	699 (77.2)	207 (22.9)	0.566	Ref.	699 (67.7)	333 (32.3)	0.380	Ref.
Male	809 (78.2)	225 (21.8)		0.92 (0.73, 1.16)	809 (66.0)	417 (34.0)		0.92 (0.75, 1.13)
**Skip breakfast**								
No	625 (74.9)	209 (25.1)	0.010	Ref.	625 (69.4)	276 (30.6)	0.034	Ref.
Yes	883 (79.8)	223 (20.2)		0.77* (0.62, 0.96)	883 (65.1)	474 (34.9)		1.10 (0.91, 1.33)
**Feeding index**								
Unhealthy	757 (78.4)	181 (21.6)	0.781	Ref.	657 (67.5)	316 (32.5)	0.559	Ref.
Healthy	834 (77.9)	237 (22.1)		1.01 (0.90, 1.13)	834 (66.4)	423 (33.7)		1.06 (0.96, 1.16)
**Walking and bicycling**								
Yes	204 (83.6)	40 (16.4.5)	0.018	Ref.	204 (70.3)	86 (29.7)	0.168	Ref.
No	1,304 (76.9)	392 (23.1)		1.50* (1.04, 2.17)	1,304 (66.3)	664 (33.7)		1.24 (0.93, 1.65)
**Sleep duration (/24 h)** ^**¶**^								
≥8	1,061 (76.1)	334 (23.9)	0.001	Ref.	1,061 (67.1)	521 (32.9)	0.261	Ref.
<8	376 (83.6)	74 (16.4)		0.64** (0.48, 0.87)	376 (64.5)	207 (35.5)		1.17 (0.95, 1.45)
**Parental characteristics**								
**Father's education (y)**								
0–5	441 (77.8)	126 (22.2)	0.026	Ref.	441 (67.6)	211 (32.4)	<0.001	Ref.
6–10	510 (74.7)	173 (25.3)		1.18 (0.89, 1.56)	510 (73.3)	186 (26.7)		0.99 (0.76, 1.30)
>10	557 (80.7)	133 (19.3)		0.89 (0.66, 1.21)	557 (61.2)	353 (38.8)		1.13 (0.87, 1.47)
**Mother's education (y)**								
0–5	476 (76.7)	145 (23.4)	0.002	Ref.	476 (68.6)	218 (31.4)	<0.001	Ref.
6–10	664 (75.5)	216 (24.6)		1.06 (0.82, 1.37)	664 (73.5)	239 (26.5)		0.99 (0.77, 1.27)
>10	368 (83.8)	71 (16.2)		0.68* (0.48, 0.96)	368 (55.7)	293 (44.3)		1.36* (1.03, 1.81)
**Mother's employment**								
No	1,211 (77.3)	356 (22.7)	0.328	Ref.	1,211 (67.8)	575 (32.2)	0.045	Ref.
Yes	297 (79.6)	76 (20.4)		0.86 (0.64, 1.15)	297 (62.9)	175 (37.1)		1.10 (0.87, 1.38)
**Parental NCDs**								
No parent with NCD	763 (74.8)	257 (25.2)	0.001	Ref.	763 (72.5)	289 (27.5)	<0.001	Ref.
One parent with NCD	509 (79.3)	133 (20.7)		0.83 (0.65, 1.06)	509 (63.5)	293 (36.5)		1.31* (1.06, 1.61)
Both parents with NCDs	236 (84.9)	42 (15.1)		0.56** (0.39, 0.80)	236 (58.4)	168 (41.6)		1.70*** (1.32, 2.19)
**Parental weight**								
Normal weight	1,057 (76.5)	325 (23.5)	0.002	Ref.	1,057 (72.5)	402 (27.6)	<0.001	Ref.
One parent overweight	363 (78.6)	99 (21.4)		0.93 (0.72, 1.22)	363 (58.3)	260 (41.7)		1.58*** (1.28, 1.95)
Both parents overweight	88 (91.7)	8 (8.3)		0.31** (0.15, 0.65)	88 (50.0)	88 (50.0)		2.10*** (1.49, 2.95)
**School characteristics**								
**School type**								
Low tuition	633 (75.8)	202 (24.2)	<0.001	Ref.	633 (71.0)	259 (29.0)	<0.001	Ref.
Medium tuition	697 (76.9)	210 (23.2)		0.96 (0.72, 1.29)	697 (74.7)	236 (25.3)		0.81 (0.54, 1.23)
High tuition	178 (89.9)	20 (10.1)		0.36*** (0.21, 0.61)	178 (41.1)	255 (58.9)		3.61***(2.33, 5.59)
**Participation in school P.A**.								
Yes	932 (78.8)	251 (21.2)	0.164	Ref.	932 (66.7)	465 (33.3)	0.928	Ref.
No	756 (76.1)	181 (23.9)		1.16 (0.92, 1.46)	576 (66.9)	285 (33.1)		1.19 (0.98, 1.46)

*PA, Physical Activity; ^¶^n = 2,574*.

### Determinants of Underweight and Overweight/Obesity Among School Children

In the multivariate analyses, after controlling for potential independent variables (school, parental, and child-level characteristics) we found that in model 3: children from high tuition schools had higher odds (AOR: 2.92, 95% CI: 1.90, 4.49) of being overweight/obese but lower odds (AOR: 0.39, 95% CI: 0.22, 0.69) of being underweight compared to children from low tuition schools. Children with parents who are overweight and suffer from NCDs respectively were more likely to be overweight/obese (AOR: 1.53, 95% CI: 1.17, 2.01 and AOR: 1.85, 95% CI: 1.30, 2.64) and less likely to be underweight (AOR: 0.62, 95% CI: 0.42, 0.93 and AOR: 0.39, 95% CI: 0.18, 0.83) compared to those with normal weight parents or parents with no NCDs, respectively. With the increasing age of the child, the likelihood for being overweight/ obese increased but for being underweight decreased. Children who were not participating in school physical activities (AOR: 1.26, 95% CI: 1.02, 1.55), had higher odds of being overweight/obese compared to children who were involved in school physical activities (Table 4).

**Table 4:** Determinants of underweight and overweight/obesity among school children.

**Characteristics**	**Normal vs. underweight**			**Normal vs. overweight/obese**		
	**Model 1**	**Model 2**	**Model 3**	**Model 1**	**Model 2**	**Model 3**
	**AOR (95% CI)**	**AOR (95% CI)**	**AOR (95% CI)**	**AOR (95% CI)**	**AOR (95% CI)**	**AOR (95% CI)**
**Child characteristics**						
**Age (in years)**						
<10	Ref.	Ref.	Ref.	Ref.	Ref.	Ref.
10–13	58*** (0.44, 0.77)	0.58*** (0.44, 0.77)	0.57*** (0.43, 0.75)	1.04 (0.83, 1.30)	1.001 (0.79, 1.26)	1.02 (0.81, 1.29)
≥14	0.67* (0.46, 0.97)	0.69 (0.47, 1.00)	0.63* (0.44, 0.92)	0.98 (0.72, 1.32)	0.87 (0.63, 1.20)	0.91 (0.66, 1.25)
**Sex**						
Female	Ref.	Ref.	Ref.	Ref.	Ref.	Ref.
Male	0.88 (0.69, 1.12)	0.91 (0.71, 1.15)	0.94 (0.74, 1.20)	0.94 (0.76, 1.17)	0.91 (0.74, 1.20)	0.90 (0.73, 1.11)
**Skip breakfast**						
No	Ref.	Ref.	Ref.	Ref.	Ref.	Ref.
Yes	0.80 (0.63, 1.01)	0.78* (0.62, 0.99)	0.80 (0.64, 1.02)	1.10 (0.90, 1.34)	1.13 (0.92, 1.38)	1.13 (0.92, 1.39)
**Feeding index**						
Unhealthy	Ref.	Ref.	Ref.	Ref.	Ref.	Ref.
Healthy	0.97 (0.76, 1.22)	0.97 (0.76, 1.23)	0.95 (0.75, 1.21)	1.09 (0.90, 1.32)	1.08 (0.88, 1.31)	1.09 (0.89, 1.32)
**Walking and bicycle**						
Yes	Ref.	Ref.	Ref.	Ref.	Ref.	Ref.
No	1.37 (0.94, 2.01)	1.36 (0.92, 1.99)	1.35 (0.91, 1.99)	1.18 (0.88, 1.58)	1.16 (0.87, 1.56)	1.09 (0.81, 1.47)
**Sleep duration (/24 h)**						
≥8 h	Ref.	Ref.	Ref.	Ref.	Ref.	Ref.
<8 h	0.70* (0.52, 0.95)	0.71* (0.53, 0.96)	0.70* (0.52, 0.95)	1.19 (0.96, 1.48)	1.17 (0.94, 1.46)	1.19 (0.95, 1.49)
**Parental characteristics**						
**Father's education**
**(years of schooling)**						
0–5		Ref.	Ref.		Ref.	Ref.
6–10		1.26 (0.88, 1.80)	1.17 (0.82, 1.67)		0.95 (0.67, 1.34)	0.99 (0.71, 1.41)
>10		1.07 (0.70, 1.63)	1.02 (0.67, 1.56)		0.91 (0.61, 1.35)	0.95 (0.64, 1.41)
**Mother's education**
**(years of schooling)**						
0–5		Ref.	Ref.		Ref.	Ref.
6–10		1.00 (0.72, 1.40)	0.95 (0.68, 1.32)		1.02 (0.73, 1.42)	1.06 (0.76, 1.48)
>10		0.75 (0.47, 1.20)	0.83 (0.53, 1.32)		1.36 (0.90, 2.06)	1.35 (0.90, 2.04)
**Mother employed**						
No		Ref.	Ref.		Ref.	Ref.
Yes		0.85 (0.62, 1.15)	0.85 (0.62, 1.15)		1.11 (0.88, 1.41)	1.10 (0.87, 1.39)
**Parental NCDs**						
None		Ref.	Ref.		Ref.	Ref.
One parent with NCDs		0.99 (0.77, 1.30)	1.02 (0.79, 1.33)		1.27* (1.02, 1.59)	1.27* (1.02, 1.59)
Both parents with NCDs		0.62* (0.42, 0.93)	0.63* (0.42, 0.93)		1.52** (1.16, 1.99)	1.53** (1.17, 2.01)
**Parental weight**						
Normal weight		Ref.	Ref.		Ref.	Ref.
One overweight parent		0.90 (0.68, 1.20)	0.94 (0.71, 1.25)		1.45** (1.16, 1.81)	1.42** (1.14, 1.78)
Both overweight parents		0.36** (0.17, 0.76)	0.39* (0.18, 0.83)		1.90*** (1.33, 2.70)	1.85** (1.30, 2.64)
**School characteristics**						
**School type**						
Low tuition			Ref.			Ref.
Medium tuition			0.93 (0.72, 1.21)			0.82 (0.56, 1.19)
High tuition			0.39** (0.22, 0.69)			2.92*** (1.90, 4.49)
**Participation in school**
**physical activities**						
Yes			Ref.			Ref.
No			1.02 (0.80, 1.30)			1.26* (1.02, 1.55)

*Model 1 included child-level characteristics, Model 2 included child-level and household/parental-level characteristics, Model 3 included child-level, household, and school-level characteristics*.

*P < 0.5, **P < 0.01, ***P < 0.001.

## Discussion

Our study identified risk factors of both overweight/obesity and underweight among school children and adolescents. This is the first known study in Bangladesh to look at a wide range of factors underlying the double burden of malnutrition based on an existing conceptual framework (15). Further, this study was conducted among urban schools with different categories of tuition fees and it addressed risk factors both within and outside of schools. This is an important study in the context of the existing double burden of malnutrition (18) and the rise of NCD-related deaths and disability in Bangladesh (19).

In our study, school type was found to be a significant predictor of overweight, obesity, and underweight, after controlling for parental and individual factors. Predictably, the proportion of overweight and obesity was higher in high tuition schools, compared to medium and low tuition schools. A study showed that resources for physical activity were better utilized in high-tuition schools in urban Dhaka, but there were opportunities for improvement (20). Resources available at high-tuition schools could be channeled to promote healthy eating and physical activity at school.The proportion of underweight was high in both low and medium tuition schools, compared to high tuition schools. In our study, school tuition was a proxy for socio-economic status of parents which indicates that children from less affluent households may not have access to adequate and nutritious food. School-based programs that address undernutrition include providing free or subsidized healthy foods for school lunch (21), particularly in low-tuition schools. In Bangladesh, the School Feeding Program implemented by the government, distributed “tetrapack” milk nutrient-fortified biscuits to all children in the intervention schools which helped increase the BMI of participating children by an average of 0.62 points compared to non-intervention schools (21). Additionally, such programs improve school attendance and performance (21). A paradoxical finding of our study is the higher prevalence of underweight in medium tuition schools, compared to low-tuition schools. Given the higher proportion of underweight among girls compared to boys in medium-tuition schools, such differences may be attributed to body image issues and eating disorders, particularly among adolescent girls (22–24). This is an interesting finding and needs to be better explored through future studies in Bangladesh.

In terms of parental characteristics, children of parents with overweight were less likely to be underweight, which demonstrates the influence of parental behaviors on children's health. A past study has shown that parental lifestyle (e.g., food habits) has a major impact on children's health (25). Among parental characteristics, children's overweight and obesity were significantly associated with their parents' overweight and NCD status. This rising burden of overweight and NCDs may be attributed to convenience foods which have become a part of the urban lifestyle (26). Children with parents with NCDs such as diabetes, cardiovascular disease, and dyslipidemia are at higher risk of developing these diseases compared to children whose parents do not have NCDs (27). Other researchers have shown that when children at risk of NCDs are overweight their chances of developing NCDs early increase significantly (7).

In terms of child-level characteristics, children were less likely to be underweight with increasing age, which may be attributed to the growth spurt and catch-up growth during puberty (28). This points to the necessity of a healthy and balanced diet at an early age rather than calorie-dense food to increase body weight. Sleeping <8 h per day was significantly associated with lower odds of being underweight, which is supported by other studies (29, 30).

Lack of physical activities was significantly associated with overweight and obesity. Similar findings have been reported from both HICs and LMICs (31). We found that children and adolescents who were physically active in and outside of school were significantly less likely to be overweight and obese. Other studies (32–34) have also emphasized the importance of creating environments that facilitate physical activity among children. Of note, the consumption of convenience foods (feeding index) was not significantly associated with overweight and obesity in our study which is different from other studies (35).

For both overweight and underweight, type of school is an important predictor (26, 32) and interventions in early childhood can support healthy lifelong habits (36). In the same population, researchers have shown that availability of healthy foods and provision of physical activities at schools differed by the type of school (26, 32). Increasing the duration and frequency of existing physical activity classes to reduce overweight and obesity (37) and making healthy and nutritious food available at school to address both overweight and underweight has been shown to be effective (38). Among children attending low-tuition schools, subsidized school lunch programs can help reduce underweight (39, 40). In many countries, programs to address overweight and underweight have focused on schools by modifying school infrastructure, training teachers, including health literacy in curricula, and drawing active involvement of parents and community members (41). Identifying children with a higher risk of malnutrition and providing them with support strategies involving parents may potentially multiply the impact of interventions by addressing the factors that contribute to the double burden of malnutrition both at home and at school (42). Despite access to resources, there are socio-cultural barriers that prevent parents from making better choices for their children in terms of physical activity and healthy food intake (20, 43). There are multiple impediments to physical activity promotion among urban school children both within and outside school in Bangladesh (20), which need to be addressed. Parents and school authorities must be made aware of the role of healthy diet and physical activity in improving cognitive function and academic success (44). It is important to develop a school-based model to support healthy eating and optimum physical activity that can be scaled up. Given the high rates of school enrolment among children and adolescents in Bangladesh, schools present an opportunity to reach a large number of students (45). A school-based model that promotes healthy foods and physical activity has a potential to address the existing double burden of malnutrition among the young Bangladeshi population.

A strength of our study is that we included a diverse set of schools which means that the findings are applicable to schools in urban Dhaka. The use of a conceptual framework allowed us to look at a wider variety of determinants than considered in previous studies of similar populations. A significant limitation of our study is that megacity Dhaka may not represent the scenario of smaller towns and rural areas of Bangladesh. Moreover, as we collected child's information from parents, there might have been some misclassification of food habits and physical activity of school children. We did not conduct any direct observation of home and school environment. Future studies should be designed to address these limitations.

## Conclusion

We found that the prevalence of overweight and obesity was higher and underweight was lower among students from high-tuition schools compared to other schools. Our findings signify that any intervention to address overweight and obesity and underweight among school children need to consider risk factors both at school and home for optimal impact. Given that overweight and underweight both lead to a range of morbidities and reduced academic performance, it is important that both policy and programmatic solutions be tested and implemented for scale-up in schools catering to different socio-economic groups to promote healthy eating and physical activity among children and adolescents across the nation. To meet the Sustainable Development Goals (SDG) for health, Bangladesh needs to achieve SDG target 2.2, which calls for “an end to all forms of malnutrition” by 2030 (46). Interventions to address malnutrition among school children will contribute to achieving multiple global goals. Reducing the double burden of malnutrition among children must be made a public health priority.

## Data Availability Statement

The raw data supporting the conclusions of this article will be made available by the authors, without undue reservation.

## Ethics Statement

The studies involving human participants were reviewed and approved by the Ethical Review Committee of International Center for Diarrhoeal Disease Research, Bangladesh (icddr, b) (Protocol Number: PR-18021). Written informed consent to participate in this study was provided by the participants' legal guardian/next of kin.

## Author Contributions

SR, MT, MR, and AH contributed to the conception and design of the study. SR, SS, MT, and AH contributed to data acquisition. MT, SR, and SS contributed to data analysis and interpretation and drafted the manuscript. MR acquired funding for this project. GS, FK, and AK reviewed and edited the document. MT and SS contributed equally to the paper and wish to share the position of the first author in the byline. All authors critically revised the manuscript, agree to be fully accountable for ensuring the integrity and accuracy of the work, and read and approved the final manuscript.

## Funding

The funding for the study was awarded to MR from the Non-Communicable Disease Program (NCDC), Directorate General of Health Services (DGHS), Ministry of Health and Family Welfare, Government of the People's Republic of Bangladesh (grant number: GR-01620). SR and AH received funding during publication stage of the manuscript from the Swedish International Development Cooperation Agency (SIDA) (GR-01455). The funders had no role in study design, data collection, and analysis, decision to publish, or preparation of the manuscript.

## Conflict of Interest

The authors declare that the research was conducted in the absence of any commercial or financial relationships that could be construed as a potential conflict of interest.

## Publisher's Note

All claims expressed in this article are solely those of the authors and do not necessarily represent those of their affiliated organizations, or those of the publisher, the editors and the reviewers. Any product that may be evaluated in this article, or claim that may be made by its manufacturer, is not guaranteed or endorsed by the publisher.
